# Metal concentrations and distributions in the human olfactory bulb in Parkinson’s disease

**DOI:** 10.1038/s41598-017-10659-6

**Published:** 2017-09-05

**Authors:** Bronwen Gardner, Birger V. Dieriks, Steve Cameron, Lakshini H. S. Mendis, Clinton Turner, Richard L. M. Faull, Maurice A. Curtis

**Affiliations:** 1 Centre for Brain Research and Department of Anatomy with Medical Imaging, University of Auckland, Auckland, New Zealand; 2 Waikato Mass Spectrometry Facility, University of Waikato, Hamilton, New Zealand; 3 Department of Anatomical Pathology, LabPlus, Auckland City Hospital, Auckland, New Zealand

## Abstract

In Parkinson’s disease (PD), the olfactory bulb is typically the first region in the body to accumulate alpha-synuclein aggregates. This pathology is linked to decreased olfactory ability, which becomes apparent before any motor symptoms occur, and may be due to a local metal imbalance. Metal concentrations were investigated in post-mortem olfactory bulbs and tracts from 17 human subjects. Iron (*p* < 0.05) and sodium (*p* < 0.01) concentrations were elevated in the PD olfactory bulb. Combining laser ablation inductively coupled plasma mass spectrometry and immunohistochemistry, iron and copper were evident at very low levels in regions of alpha-synuclein aggregation. Zinc was high in these regions, and free zinc was detected in Lewy bodies, mitochondria, and lipofuscin of cells in the anterior olfactory nucleus. Increased iron and sodium in the human PD olfactory bulb may relate to the loss of olfactory function. In contrast, colocalization of free zinc and alpha-synuclein in the anterior olfactory nucleus implicate zinc in PD pathogenesis.

## Introduction

Parkinson’s disease (PD) is expected to affect between 8.7 and 9.3 million people globally by 2030^1^, but the cause or causes of this neurodegenerative disease remain relatively unknown. Although the motor symptoms of PD are well studied, there are nonmotor symptoms that occur earlier in the disease process and may therefore provide a clue to the disease mechanisms and origins^2, 3^. One such nonmotor symptom is a decrease or complete loss of the ability to smell (hyposmia or anosmia, respectively), which occurs in over 95% of all patients^4^ and can precede a PD diagnosis by many years^5–7^. The cause of this loss of smell is unknown, but is hypothesized to relate to the early pathology that occurs in the olfactory bulbs in PD^8^.

In the PD olfactory bulb, the typical neuropathology of alpha-synuclein aggregation occurs very early in the disease process^9^, often years before diagnosis^7^. A recent, large study (*n* = 766 brains) found that the olfactory bulb was the most common sole-affected site of alpha-synuclein pathology in the brain^10^. This aggregated alpha-synuclein, in the form of Lewy bodies and Lewy neurites, is found throughout the olfactory bulb and tract but alpha-synuclein is particularly abundant in the different divisions of the anterior olfactory nucleus (bulbar, intrapeduncular, retro-bulbar and cortical)^11^. Cells are also lost in the anterior olfactory nucleus in PD, and this cell loss correlates with disease duration^11^.

Olfactory bulbs are unique in the brain in that they receive direct input from the olfactory epithelium in the nasal cavity, and so they are not protected from the outside environment by the blood-brain barrier^12–14^. The olfactory bulbs are thus especially sensitive to the uptake of environmental toxins^15^ such as metals, which may contribute to the pathology of neurodegenerative diseases, including PD^3, 16^. For example, elevated levels of iron, copper^17^, and zinc^18, 19^ cause aggregation of alpha-synuclein *in vitro*, and are taken up into the olfactory bulbs of rodents following intranasal exposure^20–22^. These metals have also been implicated in olfaction: increased olfactory bulb iron correlates with hyposmia in humans^23^, while intranasal zinc can cause anosmia in humans and animals^24–27^, and intranasal copper reduces olfaction in fish^28–30^.

However, metal concentrations have never been measured in the olfactory bulb or anterior olfactory nucleus in PD. This is despite a number of studies showing increased iron^31–37^ and decreased copper^33, 34, 38–40^ in the PD substantia nigra, which is severely degenerated in this disease. Zinc has also been reported as increased in the substantia nigra in PD^41^, although this finding is more controversial^39, 42, 43^.

**Figure 1:**
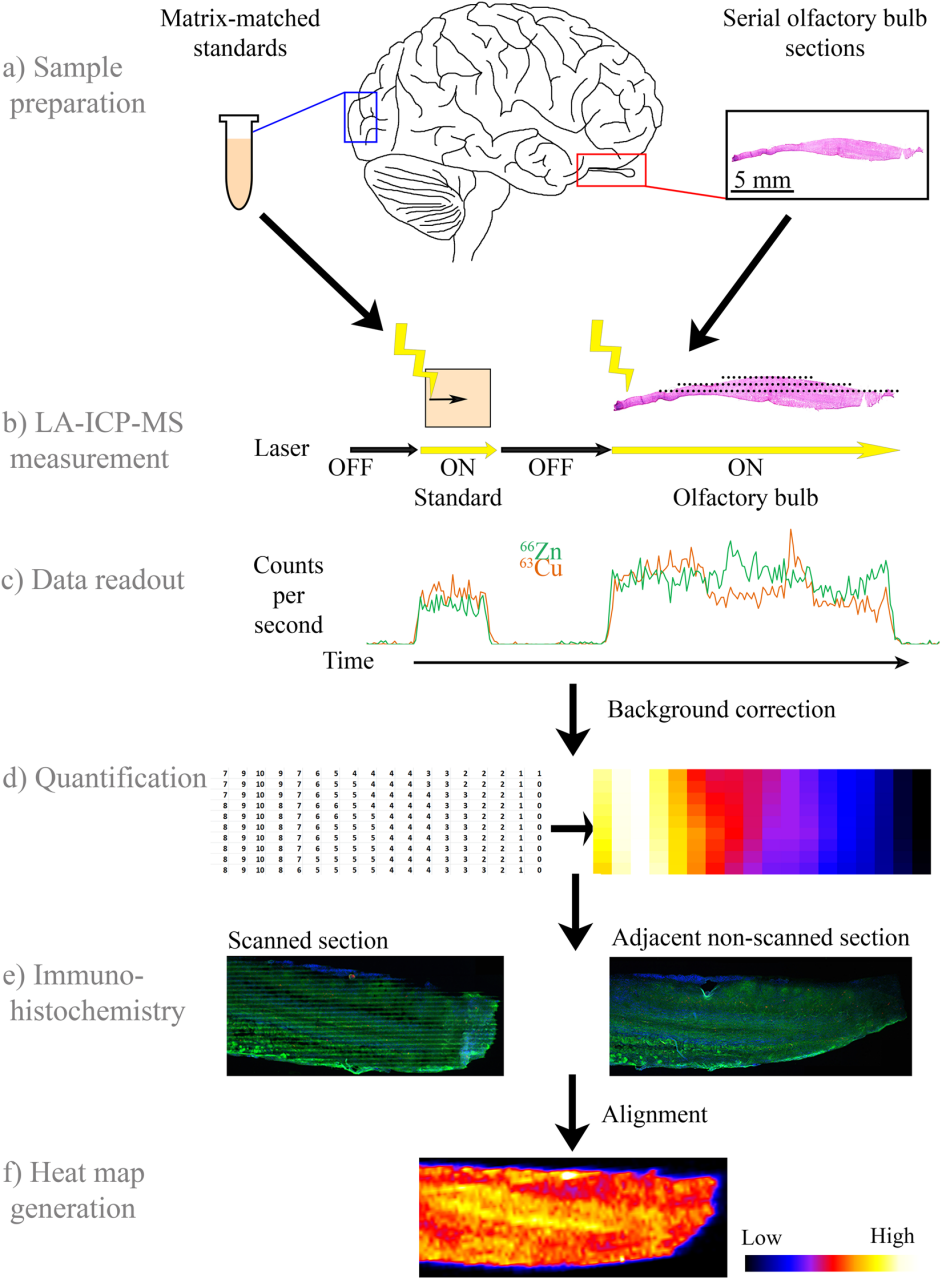
Schematic of the laser ablation inductively coupled plasma mass spectrometry (LA-ICP-MS) process. (**a**) Matrix-matched, metal-spiked standards and serial sagittal sections of olfactory bulb are cut on a cryostat and mounted onto clean glass slides. (**b**) Using LA-ICP-MS, these sections are scanned in a raster pattern and (**c**) counts of metal species are recorded over time. (**d**) Data are then background corrected and quantified using matrix-matched standard data. (**e**) Scanned and adjacent non-scanned sections are stained to determine morphology of olfactory bulb, (**f**) which enables the heat maps to be aligned and generate output.

Here we present evidence that metal concentrations are altered in the PD olfactory bulb and tract, and show for the first time by laser ablation inductively coupled plasma mass spectrometry (LA-ICP-MS) the localized patterns of iron, zinc, and copper in the human olfactory bulb, anterior olfactory nucleus, and olfactory tract (For schematic of experimental setup see Fig. 1).

## Results

### Olfactory bulb concentrations of sodium and iron are higher in PD

The weights of olfactory bulbs (normal: 71.5 ± 13.8 mg, PD: 68.8 ± 7.2 mg) and tracts (normal: 56.5 ± 13.0 mg, PD: 63.6 ± 18.0 mg) were not significantly different between the normal and PD groups. Further, no gross anatomical differences in bulb or tract structure were apparent between the two groups.

**Figure 2:**
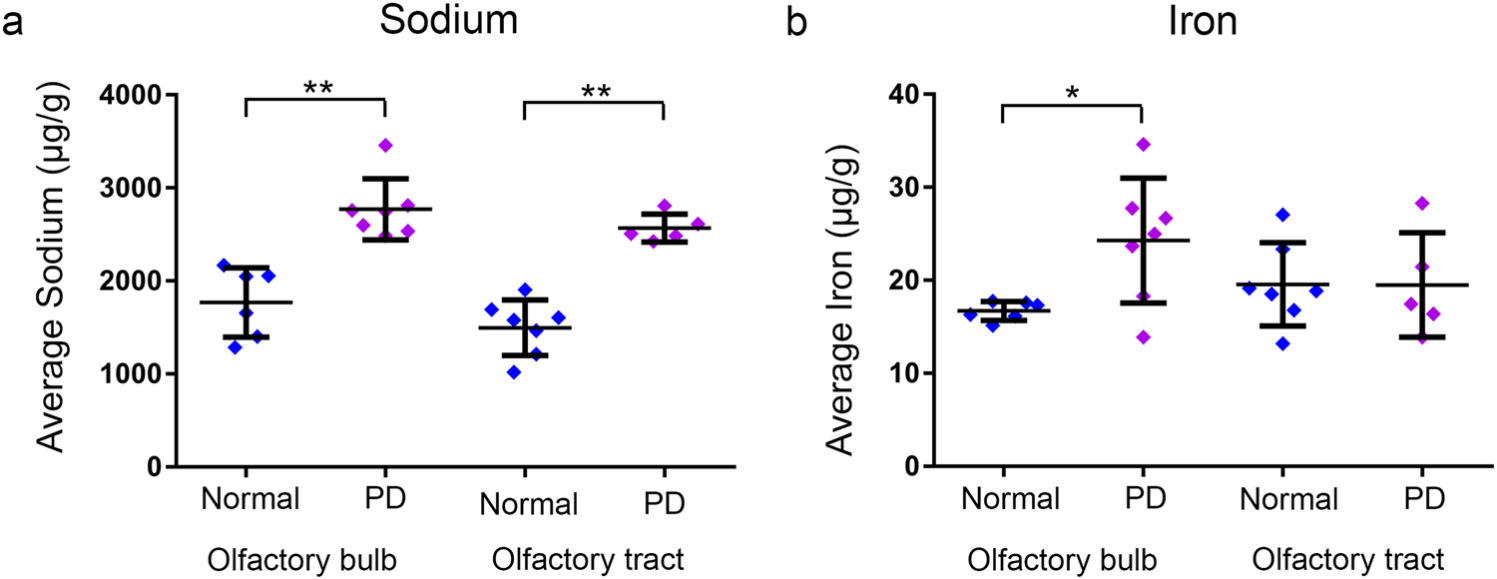
Differences in metal concentrations in the human olfactory bulb and tract in normal (*n* = 7) and Parkinson’s disease (PD; *n* = 7)) patients. (**a**) Sodium is present at higher levels in the olfactory bulb and tract in PD, while (**b**) iron is higher in the olfactory bulb in this disease. Values are mean ± SEM. *p < 0.05; **p < 0.01.

A top-down approach was used to investigate metal concentrations in the human olfactory bulb. Using inductively coupled plasma mass spectrometry (ICP-MS), which measures metal concentrations in homogenized tissue samples, a range of metals were detected in the olfactory bulb and tract (Table 1). Sodium and potassium were present at mg/g levels, while iron, copper, calcium, magnesium, zinc, and rubidium were detected at µg/g concentrations. Nickel, chromium, manganese, lead and vanadium were found at trace levels, of less than 1 µg/g on average. Aluminium, arsenic, selenium, strontium, cadmium, and barium were not detected or were present at levels below their respective limit of detection (LoD).

**Table 1:** Metal concentrations in the human olfactory bulb and tract in normal (*n* = 4) and Parkinson’s disease (PD; *n* = 4) patients. Values are mean ± SEM, wet weight. LoD: Limit of detection (3 σ^*blank*^); LoQ: Limit of quantification (10 σ^*blank*^).

Element	LoD (µg/g)	LoQ (µg/g)	Parkinson’s disease		Normal	
			Olfactory bulb (µg/g)	Olfactory tract (µg/g)	Olfactory bulb (µg/g)	Olfactory tract (µg/g)
^23^Na Sodium	0.0091	0.0302	2,770.74** ± 123.96	2,567.88** ± 66.99	**1,768.34 ± **152.67	**1496.32 ± **112.69
^54^Fe Iron	0.0168	0.0560	24.27* ± 2.53	19.49** ± **2.51	**16.70 ± **0.41	19.55** ± **1.69
^63^Cu Copper	0.0001	0.0003	1.59** ± **0.119	1.01** ± **0.205	1.64** ± **0.168	1.29** ± **0.396
^39^K Potassium	0.0060	0.0199	1,827.89** ± **271.60	1,851.38** ± **224.57	1,534.15** ± **141.87	2,031.56** ± **256.64
^43^Ca Calcium	0.0342	0.1141	438.44** ± **102.34	476.03** ± **174.13	262.43** ± **140.54	227.87** ± **48.43
^24^Mg Magnesium	0.0090	0.0301	74.58** ± **4.78	76.43** ± **3.84	60.78** ± **2.41	64.74** ± **3.65
^68^Zn Zinc	0.0008	0.0027	19.04** ± **5.37	40.46** ± **10.60	31.92** ± **9.95	25.62** ± **7.56
^85^Rb Rubidium	0.0000	0.0000	1.455** ± **0.100	1.686** ± **0.180	1.776** ± **0.202	2.301** ± **0.213
^60^Ni Nickel	0.0001	0.0004	0.260** ± **0.102	0.900** ± **0.379	0.536** ± **0.201	0.351** ± **0.141
^52^Cr Chromium	0.0003	0.0010	0.261** ± **0.020	0.331** ± **0.026	0.2 44** ± **0.023	0.249** ± **0.031
^55^Mn Manganese	0.0001	0.0003	0.179** ± **0.013	0.185** ± **0.015	0.169** ± **0.007	0.147** ± **0.013
^207^Pb Lead	0.0001	0.0003	0.062** ± **0.025	0.137** ± **0.053	0.096** ± **0.021	0.063** ± **0.033
^51^V Vanadium	0.0001	0.0002	0.077** ± **0.005	0.096** ± **0.009	0.077** ± **0.008	0.070** ± **0.010

*Differ between PD and normal groups (*p* < 0.05); **differ between PD and normal groups (*p* < 0.01).

The average sodium concentration (Fig. 2), however, was 57% higher in the PD olfactory bulb (*p* < 0.01) and 71% higher in the PD olfactory tract (*p* < 0.05; Fig. 2a) compared to normals. In addition, iron was 25% higher overall in the PD olfactory bulb (*p* < 0.05; Fig. 2b) compared to the normal group. All metals were detected at similar concentrations in the olfactory bulb and tract using ICP-MS. Most metal concentrations were similar between PD and normal groups. No metals were significantly correlated with post-mortem delay, and there was no relationship between age or sex and any metal concentrations.

### Copper, zinc, and iron are differentially expressed in olfactory bulb layers

**Figure 3:**
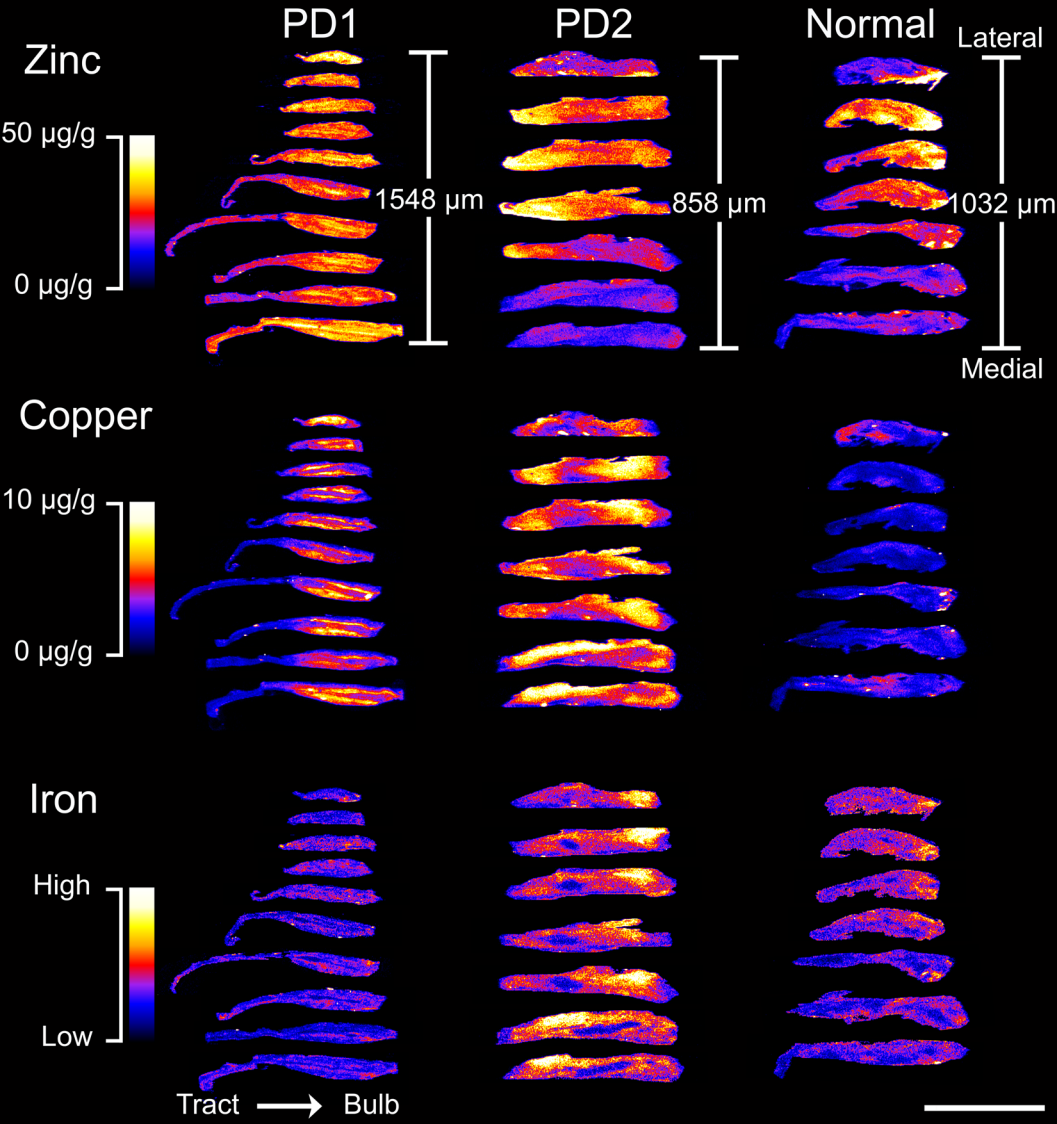
Heat maps of zinc, copper, and iron concentrations in serial sagittal sections of olfactory bulbs and tracts. Tissue from three human subjects was scanned using laser ablation inductively coupled plasma mass spectrometry (LA-ICP-MS) to produce these heat maps. Lateral distance from the first scanned section to the last scanned section for each case were 1548 µm, 858 µm, and 1032 µm for PD1, PD2, and Normal cases, respectively. Scale bar: 1 cm.

LA-ICP-MS can accurately measure metals in biological tissue at concentrations of less than 1 µg/g^44^ at spatial resolutions of approximately 1–100 µm^45, 46^. This technique was used to investigate the distributions of zinc, copper, and iron in serial sections of olfactory bulb and tract from one normal and two PD subjects, at a resolution of 50 µm. Although sodium was also of interest, accurate measurements of this metal could not be obtained using the current experimental setup because of the very high levels of this element relative to brain regions used for optimization experiments. Resulting heat maps (Fig. 3) showed consistent zinc, copper, and iron concentrations over serial scans from each bulb, and metal distribution patterns were similar in the three olfactory bulbs, although concentrations differed.

**Figure 4:**
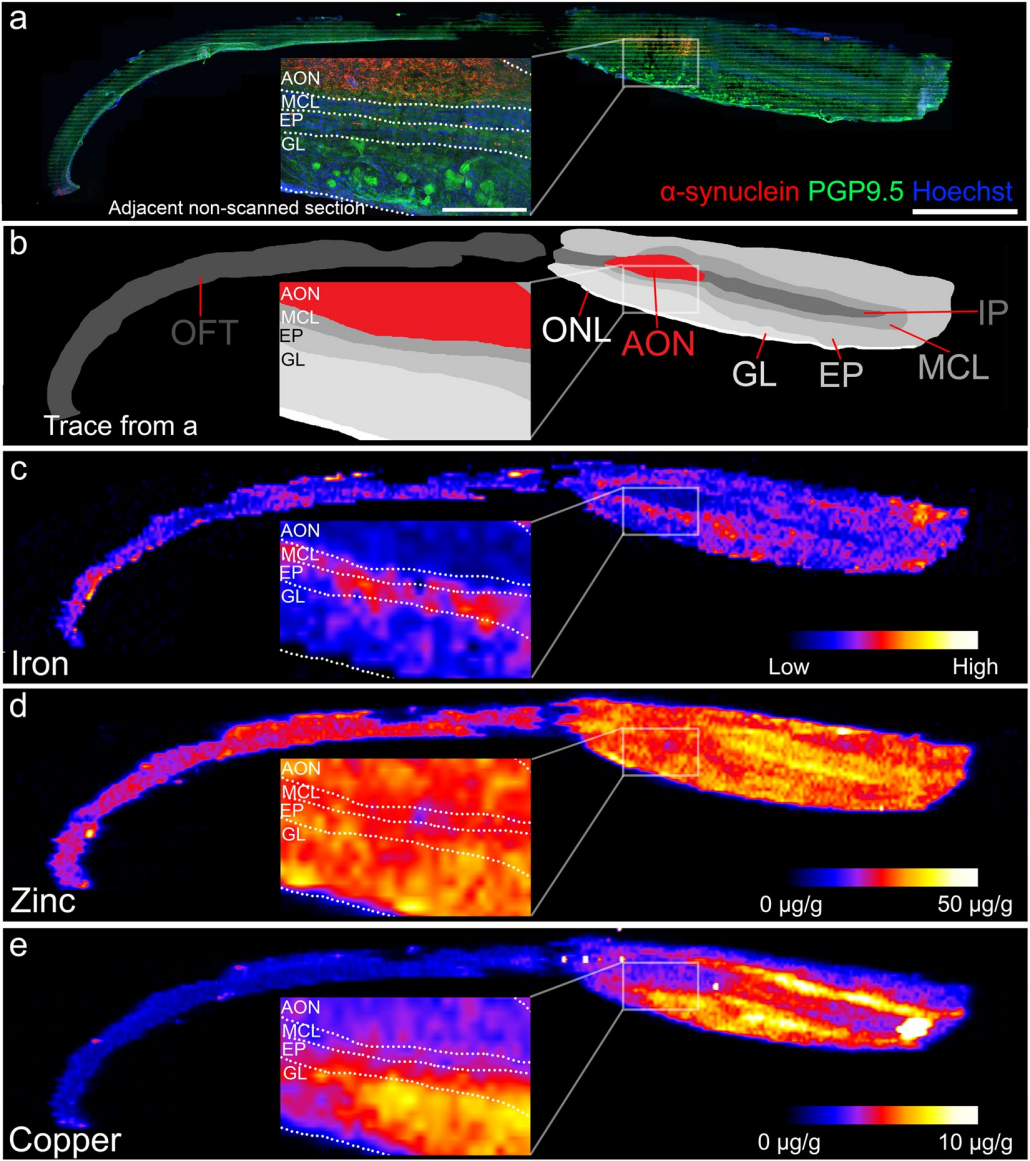
Micrographs and heat maps of a single section from case PD1. Note the high levels of aggregated alpha-synuclein in the anterior olfactory nucleus; this region is low in both iron and copper, but high in zinc. (**a**) Overview of scanned section stained with phosphorylated alpha-synuclein (red), PGP9.5 (green), and Hoechst (blue). Inset shows anatomical detail of the anterior olfactory nucleus and the mitral cell, external plexiform, and glomerular layers in an adjacent section. (**b**) Trace from (**a**) showing the distinct layers of the olfactory bulb. Zoom is shown inset. (**c**–**e**) Heat maps of localized iron (**c**), zinc (**d**), and copper (**e**) concentrations in the olfactory bulb and tract, with zooms shown inset. OFT: Olfactory tract; ONL: Olfactory nerve layer; AON: Anterior olfactory nucleus; GL: Glomerular layer; EP: External plexiform; MCL: Mitral cell layer; IP: Internal plexiform and granule cell layer. Scale bars: 2 mm (**a**–**e**) and 500 µm (**a**–**e** inset)

In aged humans, the olfactory bulb layers become thinner^47^ and are often less well organized than in younger bulbs^48^. This was observed to varying degrees in the three olfactory bulbs that were investigated using LA-ICP-MS and immunohistochemistry. PD1 had very defined olfactory bulb layers, while PD2 and the normal brain displayed much less organized layers that were more difficult to define. PD1 was thus used for detailed investigation of metal localization in the olfactory bulb.

The olfactory bulb from PD1 (Fig. 4) was immunohistochemically labelled with protein gene product 9.5 (PGP9.5), which labels neurons, and phosphorylated alpha-synuclein, which labels Lewy bodies and neurites (Fig. 4a). For the ICP-MS experiments, we increased the numbers to 7 PD patients and 7 normal patients. The combination of this staining with Hoechst, which labels cell nuclei, allowed the layers of the olfactory bulb to be traced (Fig. 4b). Immunohistochemically labelled adjacent non-scanned sections confirmed these layers. By overlaying traces onto LA-ICP-MS heat maps, iron was very low in the internal plexiform and granule cell layer (Fig. 4c), while zinc was highest in these layers (Fig. 4d). Iron concentrations were low in the anterior olfactory nucleus, but was present at highest levels in the mitral cell and external plexiform (Fig. 4c), and copper was highest in the external plexiform and mitral cell and glomerular layers (Fig. 4e).

### Zinc is present in anterior olfactory nucleus neurons that contain aggregated alpha-synuclein

**Figure 5:**
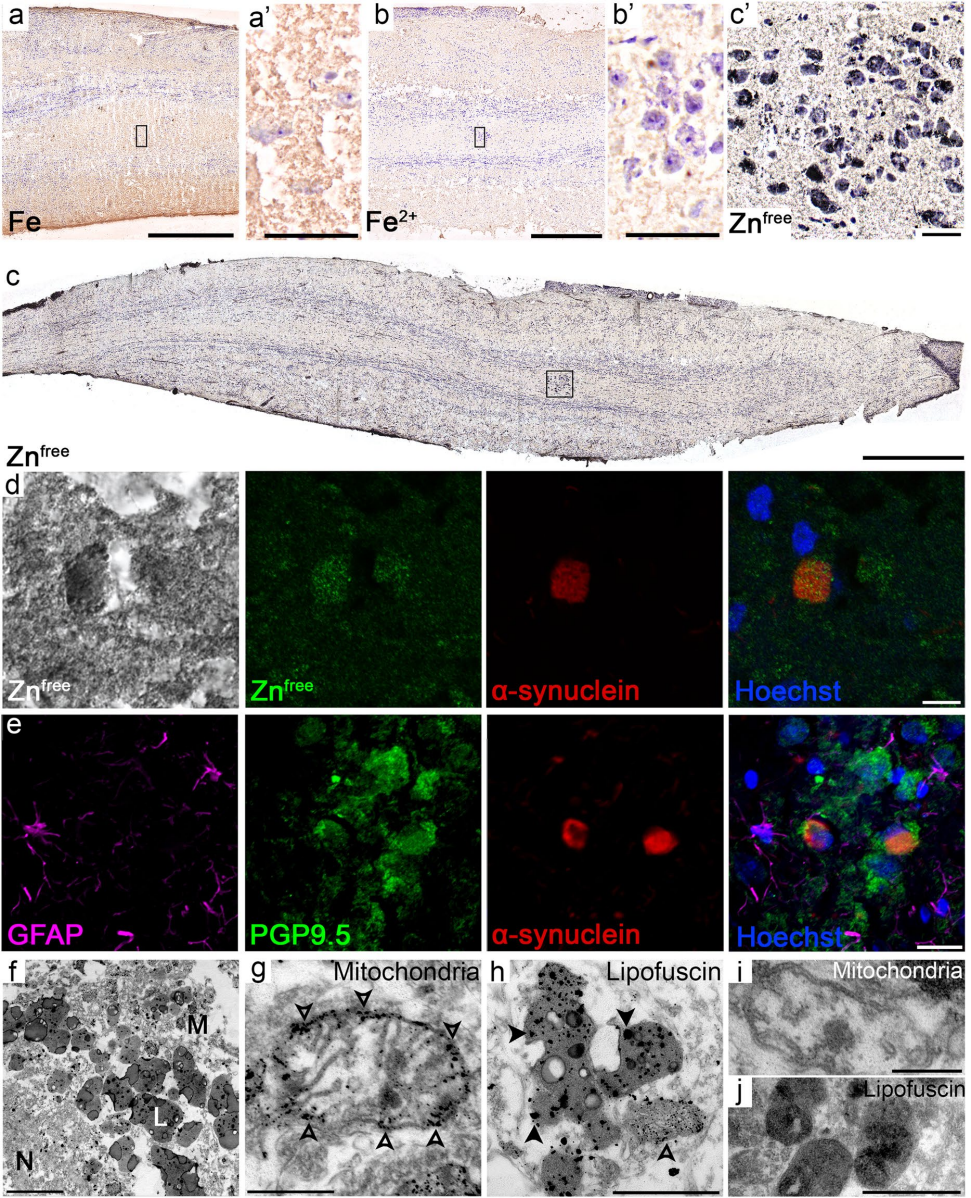
Iron and zinc histological stains in the anterior olfactory nucleus in the Parkinson’s disease (PD) olfactory bulb. (**a)** Nonheme iron (brown) is present predominantly in the outer layers of the olfactory bulb and tract. Cell nuclei and neuronal bodies are counterstained with cresyl violet. (**a’**) Zoom of boxed region in (**a**) showing very little nonheme iron in anterior olfactory nucleus cells. (**b**) Ferrous iron (brown) is present at low levels in the olfactory bulb and tract. Cell nuclei and neuronal bodies are counterstained with cresyl violet. (**b’**) Zoom of boxed region in (**b**) showing very little ferrous iron in anterior olfactory nucleus neurons. (**c**) Free and loosely bound zinc (black and brown) is present throughout the olfactory bulb and tract. Cell nuclei and neuronal bodies are counterstained with cresyl violet. (**c’**) Zoom of boxed region in (**c**) showing multiple zinc granules in anterior olfactory nucleus neurons. (**d**) Light micrograph from the anterior olfactory nucleus showing free and loosely bound zinc (black), followed by confocal micrographs of the same cell showing that the free zinc (green, using reflected light microscopy) is located within large aggregates of phosphorylated alpha-synuclein (red). (**e**) Confocal micrographs showing phosphorylated alpha-synuclein (red) aggregates in cells that are positive for PGP9.5 (green), but not GFAP (purple), in the anterior olfactory nucleus. (**f**) Transmission electron micrograph of free zinc staining in the anterior olfactory nucleus. Cells in this region were rich in lipofuscin pigment (L) and mitochondria (M), both of which contained numerous silver granules (corresponding to free zinc). The cell nucleus (N) is also visible in this micrograph. (**g**) Small silver granules (open arrowheads) were common on mitochondria in cells in the anterior olfactory nucleus. (**h**) Lipofuscin pigment contained silver granules that were often very large (closed arrowheads). Smaller silver granules can also be seen in a mitochondrion in this image (open arrowhead). (**i**) Mitochondria and (**j**) lipofuscin did not contain silver granules under negative control conditions, where no sodium sulfide was used. Scale bars: a, b: 500 µm; a’, b’: 50 µm; c: 1 mm; c’: 40 µm; d: 10 µm; e: 20 µm; f: 2 µm; g: 500 nm; h: 1 µm; i: 250 nm; j: 1 µm.

In all PD olfactory bulb sections, there was a region of dense phosphorylated **(**Figs 4 and 5 **)** alpha-synuclein aggregates (Fig. 4a), which corresponds to a rostral region of the anterior olfactory nucleus (Fig. 4b). This region was also seen in the olfactory tract in some sections (data not shown). Iron was detected at relatively low levels in the PD anterior olfactory nucleus using LA-ICP-MS (Fig. 4c). Nonheme ferric and ferrous iron can also be visualized using the DAB-enhanced Perls and Turnbull histological stains; the Perls stain allows visualization of mostly ferric iron, while the Turnbull stain is specific for ferrous iron^49^. Although there was positive staining in most layers of the olfactory bulb (Fig. 5a,b), the anterior olfactory nucleus displayed very little cell-specific staining for both ferric and ferrous iron (Fig. 5a,b).

While copper was also very low in the anterior olfactory nucleus (Fig. 4e), zinc was present at relatively high concentrations (approximately 20–30 µg/g) in this region (Fig. 4d). The immersion neoTimm autometallographic stain is a sensitive and specific method for visualizing free and loosely bound zinc in tissue^50^. Using this method, an intense, granular staining pattern could be observed in the large neurons of the anterior olfactory nucleus (Fig. 5c,c). When combined with immunofluorescence techniques, zinc was observed within large aggregates of phosphorylated alpha-synuclein (Fig. 5d). Zinc was also observed in cells that did not contain alpha-synuclein (Fig. 5d). These aggregates of phosphorylated alpha-synuclein occurred in cells that were positive for protein gene product 9.5 (PGP9.5), a neuronal marker, but not glial fibrillary acidic protein (GFAP), an astrocytic marker (Fig. 5e).

Using transmission electron microscopy, silver enhancement of zinc could be observed in large cells of the anterior olfactory nucleus that contained high loads of large lipofuscin pigments (Fig. 5f). In both the normal and PD anterior olfactory nucleus, there were many large silver granules in the lipofuscin (Fig. 5g), while multiple smaller silver granules could also be observed in mitochondria (Fig. 5h). This pattern of silver granule staining was present throughout all olfactory bulb layers, although there was much less lipofuscin in other regions, and the granules were smaller. These silver granules were not present in any regions or organelles in the negative control condition, where the sodium sulfide step was omitted (Fig. 5i,j).

## Discussion

In the present study, increased concentrations of iron and sodium were detected in the PD olfactory bulb. Even though, the anterior olfactory nucleus is the main site of aggregated alpha-synuclein pathology in the PD olfactory bulb^9, 51^, iron was present at very low levels in this region. Copper was also low in the anterior olfactory nucleus, but zinc was relatively high in this secondary olfactory processing region. In addition, free zinc, which is neurotoxic at high levels^52^, was present in cell soma and Lewy bodies in the anterior olfactory nucleus.

The range of metal concentrations that were measured from both normal and PD olfactory bulbs and tracts in the current study were similar to those previously reported in rats, although exact concentrations varied between both previous investigations and the current study. Both sodium and potassium had levels above 800 µg/g in the combined olfactory bulb and tract in rats, while magnesium, calcium, iron, and zinc were present at between 10 and 200 µg/g (wet weight)^53, 54^. Concentration ranges were also similar to a previous study in the human olfactory bulb, tract, and trigone^55^. The findings from the current investigation therefore highlight the similarities in olfactory bulb and tract metal concentrations between humans and rodents, and confirm the reproducibility of ICP-MS as a technique. However, although the current study aligns closely with these previous studies, which were all performed on unfixed tissue, results were markedly different in two other studies that used chemically fixed olfactory bulbs and tracts from cadavers that had previously been used for medical student teaching^56, 57^. The combination of these results underscore the importance of tissue preparation, as ICP-MS is very susceptible to contamination^58–60^.

The findings of increased iron and sodium in PD olfactory bulbs, and increased sodium in PD olfactory tracts, are notable because both metals are known to be tightly regulated in healthy tissue^61, 62^. In contrast, both iron and sodium are elevated in affected brain regions in pathological conditions such as Alzheimer’s disease^63–66^, Huntington’s disease^66–69^, multiple sclerosis^70–73^, and tumors^74–76^. Iron is also elevated in the PD substantia nigra^31–37^, although no changes in sodium have been reported in this disease^43^. It could thus be that sodium and iron accumulation are general markers of pathology, rather than specific to PD. However, this increase in iron and sodium may be clinically important in producing the hyposmia and anosmia that occur in PD. Neurodegeneration disorders with brain iron accumulation result in increased olfactory bulb iron and decreased olfactory ability^23^, and iron is known to be essential for the function of enzymes that are important in normal olfaction, such as neuronal nitric oxide synthase and hydroxyanthranilic acid^77^. Sodium also plays a role in olfaction: voltage-gated sodium channels are necessary for odor perception in mice, fruit flies, and humans^78, 79^.

The heat maps from LA-ICP-MS data show consistent patterns and concentrations of zinc, copper, and iron across serial sections of olfactory bulbs. This consistency over scans confirms that LA-ICP-MS is a sensitive, reproducible technique, as has been previously reported^80^, and demonstrates that metal concentrations are maintained in defined regions throughout the human olfactory bulb, like in mice^81^. The distribution of some metals was also similar to that seen in the mouse^81^: zinc had the most homogeneous distribution, while copper was more localized and had high concentrations in the glomerular layer. However, in the human, copper was also high in the external plexiform and mitral cell layer, unlike in the mouse olfactory bulb^81^. Iron distribution was also different between the human and mouse olfactory bulb: in humans, the internal plexiform and granule cell layer were very low in iron, while in the mouse these regions were relatively high. In addition, iron was lowest in the mouse external plexiform^81^, while the human external plexiform had one of the highest iron concentrations. These findings highlight the importance of using human tissue, as this and other studies^82–84^ have uncovered differences in the structure and function of the olfactory bulb between humans and rodents.

The LA-ICP-MS heat maps were particularly interesting because metal concentrations in the human anterior olfactory nucleus could be measured for the first time. The anterior olfactory nucleus undergoes a number of pathological changes early in PD, the most prominent being the aggregation of alpha-synuclein as Lewy bodies and Lewy neurites^9^. In the present study, the anterior olfactory nucleus was easily identified in PD patients using immunohistochemistry for phosphorylated alpha-synuclein, with both PD olfactory bulbs showing intense staining in this region. It was anticipated that the anterior olfactory nucleus would be high in iron, especially in the PD patients; previous studies of the PD substantia nigra, which contains a high load of aggregated alpha-synuclein, have reported an increase in this metal^31–37^, including in individual surviving dopaminergic neurons in this region^85^. However, the anterior olfactory nucleus contained remarkably low levels of iron relative to the rest of the olfactory bulb in both PD and normal patients, and was also low in nonheme ferrous and ferric iron in this region. Although copper concentrations in the anterior olfactory nucleus were also low, zinc concentrations were relatively high, and were thus investigated further.

The neoTimm stain was used to label free and loosely bound zinc, which is considered to be more neurotoxic than bound forms of zinc^86^, and which is increased in cells in conditions of oxidative stress^87^. While earlier forms of this histological technique were not specific for zinc ions, more recent iterations have been validated as specific and sensitive for zinc^88^. In the current study, zinc stains in the human olfactory bulb showed high zinc throughout the olfactory bulb and tract, similar to previous histological studies in the mouse olfactory bulb^89, 90^, and similar to LA-ICP-MS studies in the human (in the present study) and mouse^81^.

In both normal and PD patients, large cells in the anterior olfactory nucleus contained coarse, darkly stained granules in their soma using the neoTimm stain. When combined with immunofluorescence, free or loosely bound zinc was observed within Lewy bodies in anterior olfactory nucleus neurons. Alpha-synuclein interacts with zinc^19^ and forms sodium dodecyl sulfate (SDS)-resistant dimers^18^ *in vitro*, which may explain the presence of zinc in alpha-synuclein aggregates in the current study. In addition, using TEM, the majority of free zinc in both the PD and normal anterior olfactory nucleus was observed in lipofuscin. These lipofuscin pigments are visible using transmission electron microscopy as electron-dense inclusions that also contain electron-lucent components^91^. Because lipofuscin pigments are known to contain zinc in healthy tissue^92^ and increase with age^91^, this zinc in the anterior olfactory nucleus may therefore be a result of the normal aging process. Olfactory ability declines in healthy humans from approximately 60 years of age^47, 93^, and this may be related to the accumulation of zinc and lipofuscin in anterior olfactory nucleus cells, which are important in secondary olfactory processing^11^. Additionally, intranasal zinc causes hyposmia and anosmia in humans^24–26^ and is commonly used to experimentally ablate olfaction in animal models^27^, lending support to this idea that olfactory bulb zinc causes olfactory dysfunction. This somatic free and loosely bound zinc may also make cells in the anterior olfactory nucleus more vulnerable to the aggregation of alpha-synuclein in early PD, especially within lipofuscin: alpha-synuclein-positive particles and small Lewy bodies have been detected within lipofuscin pigment in the PD brain stem^94^, although this has not been described in the olfactory bulb.

In the current study, mitochondria in the anterior olfactory nucleus also contained free or loosely bound zinc. Mitochondrial uptake of zinc has been previously reported in neurons *in vitro* and can contribute to oxidative stress in these cells by causing mitochondrial dysfunction and reactive oxygen species production^87, 95–98^. Thus, mitochondrial free zinc may contribute to the oxidative stress that has been implicated in the PD pathological process^41, 99^ through its role in mitochondrial dysfunction.

In summary, while iron and sodium are increased in the PD olfactory bulb and may be involved in the olfactory dysfunction that occurs in this disease, iron is present at low levels in regions of alpha-synuclein pathology. Zinc, however, is high in the PD anterior olfactory nucleus, including within Lewy bodies. Free zinc in this region may contribute to olfactory dysfunction and typical Lewy body pathology in PD, possibly through oxidative stress.

## Materials and Methods

### Human brain tissue

Human olfactory bulb and tract tissue was obtained from the Neurological Foundation Douglas Human Brain Bank. Tissue was acquired with the full informed consent of families and this process was approved by the University of Auckland Human Participants Ethics Committee (Reference Number 011654). All methods were performed in accordance with the relevant guidelines and regulations. All PD patients had a clinical diagnosis of PD in life and neuropathologic findings consistent with a diagnosis of PD. Tissue was chosen for inclusion based on a combination of clinical diagnosis and post-mortem pathology. All brains were analysed by a neuropathologist. The Lewy pathology in PD patients was staged according to the method of the BrainNet Europe consortium^100^. Most PD cases had some amyloid and the occasional evidence of tau in keeping with age related changes. Only PD34 had plausible Alzheimer’s pathology. We confirmed in our results that PD34 was not skewing our data, which gave us confidence that we were truly studying PD-related changes. PD1 and PD2 that were used for the LA-ICPMS had no evidence of amyloid or tau.

For inductively coupled plasma mass spectrometry (ICP-MS) experiments, tissue was taken from seven neurologically normal patients and seven PD patients (Table 2). All normal and PD cases were sex-and age-matched (4 females and 3 male in each group; average age: normal 69 ± 8.7 years, PD 76.5 ± 9.2 years). There was also no significant difference in post-mortem delay between groups (normal 18.1 ± 7.7 h, PD 13.1 ± 2.9 h). Olfactory bulbs and tracts were dissected from the brain, snap frozen using CO_2_ powder, and stored at −80 °C until required.

**Table 2:** Case details of olfactory bulb tissue. Parkinson’s disease (PD), Cortical Lewy body disease (CLBD), Alzheimer’s disease (AD), *detailed pathological description of this case can be found in material and methods.

Experimental	Case type	Pathology	Case	Age (years)	Sex	Post-mortem delay (h)	Cause of death
usage							
ICP-MS	Normal	Normal		82	Female	27.5	Myocarditis
ICP-MS	Normal	Normal		72	Male	12	Stab wound to hart
ICP-MS	Normal	Normal		73	Female	12	Metastatic cancer
ICP-MS	Normal	Normal		61	Female	22	Ischaemic heart disease
ICP-MS	Normal	Normal		80	Male	19	Ischaemic heart disease
ICP-MS	Normal	Normal		51	Male	18	Ischaemic heart disease
ICP-MS	Normal	Normal		63	Female	16	Dissecting aortic aneurism
ICP-MS	Parkinson’s disease	PD/CLBD/early AD	PD34	75	Female	10	Cerebrovascular accident
ICP-MS	Parkinson’s disease	PD/CLBD	PD35	73	Male	16	Pneumonia
ICP-MS	Parkinson’s disease	PD/CLBD	PD58	82	Female	18	Unknown
ICP-MS	Parkinson’s disease	PD/CLBD	PD43	60	Female	16	Bronchopneumonia and multiple organ failure
ICP-MS	Parkinson’s disease	PD	PD73	83	Female	4	Pneumonia
ICP-MS	Parkinson’s disease	PD/CLBD	PD60	80	Male	18	Urosepsis
ICP-MS	Parkinson’s disease	PD	PD41	81	Male	13	Renal failure/urinary sepsis
LA-ICP-MS	Normal (stroke)	Normal	Normal	80	Male	17	Bronchopneumonia
LA-ICP-MS	Parkinson’s disease	PD/CLBD	PD1	91	Female	5	Parkinson’s disease
LA-ICP-MS	Parkinson’s disease	PD/CLBD	PD2	65	Male	17	Bronchopneumonia

For laser ablation ICP-MS (LA-ICP-MS) and histological experiments to investigate zinc, copper, and iron distribution in the olfactory bulb and tract, tissue was taken from one neurologically normal subject (male, 80 years, post-mortem delay 17 h) and two PD patients (PD1: female, 91 years, post-mortem delay 5 h; male, PD2: 65 years, post-mortem delay 17 h). For the normal subject, cause of death was a stroke in the posterior cerebral artery, which mainly affected the occipitotemporal cortex. Since the stroke and its effects were far from the olfactory bulb and tract and did not affect the anterior cerebral or medial fronto-basal arteries, a “normal” classification relating to the olfactory and frontal regions of the brain was given.

### ICP-MS analysis

All equipment used for ICP-MS experiments was acid washed in 30% HNO_3_ overnight before use. Frozen olfactory bulb blocks were divided into bulb and tract regions and weighed. Tissue was first digested in 600 µL of concentrated HNO_3_ (69%, Merck) at room temperature overnight, and then at 80 °C for 30 min. Following cooling, 300 µL of concentrated H_2_O_2_ (30%, Merck) was added and the tissue was further digested for 4 h at room temperature, and then for 15 min at 70 °C. Samples were diluted to 2% HNO_3_ and filtered, prior to analysis using a SCIEX ELAN DRC II ICP-MS (PerkinElmer). A total of three replicates were measured from each sample, and a set of blank control (reagents only) replicates was measured to correct for contamination during processing. Settings for the ICP-MS were as follows: Radio-frequency power 1400 W, nebulizer gas flow (Ar) 0.86 L min^−1^, auxiliary gas flow (Ar) 1.2 L min^−1^, plasma gas flow (Ar) 15 L min^−1^. The ICP-MS was calibrated for iron, calcium, potassium, and sodium using single-element standards (140-051-265, −115, −205, and −195; SCP Science), combined and diluted to an overall concentration of 10 µg/mL of each metal. All other metals were calibrated using a multi-element standard (IV-ICPMS-71A, Inorganic Ventures), diluted to a 0.05 µg/mL concentration. A certified reference material of river water (SLRS-5, National Research Council of Canada) was measured at the beginning of the ICP-MS experiment to evaluate the quality of the data. The ICP-MS was tested for carryover every eight samples, and the probe was rinsed in 2% HNO_3_ between each sample, while calibration standards were remeasured every 24 samples to correct for machine drift. The high fat content of brain (approximately 40% in grey matter, 50–65% in white matter, and 80% in myelin^101^) meant that common biological certified reference materials such as bovine liver (approximately 11% fat^102^) were unsuitable for use as a measure of digestion efficiency. Final concentrations from ICP-MS measurements thus provide a guide to total concentrations rather than absolute quantities. Limits of detection (LoD) and limits of quantification (LoQ) are given in Table 1. The LoD was calculated using the standard deviation of the blank (σ^*blank*^) as LoD = 3σ^*blank*^, and LoQ was calculated as LoQ = 10σ^*blank*^. Sodium, iron, copper, potassium, calcium, magnesium, zinc, rubidium, nickel, chromium, manganese, lead, vanadium, and cesium were present at levels above their LoD and LoQ. In contrast, although aluminium, arsenic, selenium, strontium, cadmium, and barium were also measured, the detected levels were below the LoDs for these metals, and so were not included in the results.

### Statistical analysis

Data from ICP-MS were normally distributed (D’Agostino & Pearson omnibus normality test), so unpaired, two-tailed *t* tests and Pearson correlation coefficients were performed using GraphPad Prism v6.05. The level of significance was set at *p* = 0.05 and values are given as mean ± SEM, in µg/g (wet weight).

### LA-ICP-MS imaging

A schematic representation of these methods can be seen in Fig. 1. For absolute quantification of copper and zinc, matrix-matched standards were made; iron-spiked standards were also trialed but the homogenization of iron was unsuccessful. Tissue from the visual cortex of a human brain (female, 80 years, 30 h post-mortem delay) was manually homogenized using a PTFE-coated blade. 500 mg of tissue homogenate per standard was spiked with 30 µL of varying concentrations of copper and zinc salts (copper(II) nitrate hydrate and zinc nitrate; >99.999% purity; Sigma Aldrich) made up in MilliQ water. Final added concentrations (wet weight) of metals in the matrix-matched standards were as follows: 0 µg/g, 0.5 µg/g, 5 µg/g, 10 µg/g, 20 µg/g, 40 µg/g (copper only), 50 µg/g (zinc only). Following the addition of metal solutions, tissue was further homogenized in a Bullet Blender homogenizer (Next Advance) and snap frozen in histology molds. Matrix-matched standards, like other tissue used for LA-ICP-MS experiments, were sectioned at 30 µm on a cryostat (CM3050, Leica Biosystems), mounted onto acid-washed slides, and air dried.

To confirm a linear relationship between added metal salts and measured ion intensity, matrix-matched standards were scanned using LA-ICP-MS, and R^2^ values of 0.9954 (copper) and 0.9936 (zinc) were obtained (Suppl. Figure 1). To reduce overall experimental time, only the 0 µg/g and 20 µg/g matrix-matched standards were used for quantification in further experiments.

Olfactory bulb and tract sections were scanned using a RESOlution 193 excimer laser ablation system (Australian Scientific Instruments) coupled to a SCIEX ELAN DRC II ICP-MS (PerkinElmer). The ICP-MS was calibrated each day using uranium and thorium ion intensities from standard reference material 612 (National Institute of Standards and Technology). Operational conditions and experimental parameters for LA-ICP-MS can be found in Tables 3 and 4. For accurate quantification of copper and zinc, and accurate relative quantification of iron, matrix-matched standards were measured at the beginning and end of each LA-ICP-MS scan, as well as approximately every 30 min during scans. Background measurements (laser off) were also taken both before and after each scanned line to correct for machine drift.

**Table 3:** Operational conditions used for LA-ICP-MS experiments.

Sample introduction		System parameters	
Radio-frequency power	1350 W	Laser ablation system	RESOlution
Nebulizer gas flow (Ar)	0.6 L min^−1^	Ablation mode	Line scans
Auxiliary gas flow (Ar)	1.2 L min^−1^	Wavelength	193 nm
Plasma gas flow (Ar)	15 L min^−1^	Repetition frequency	5 Hz
Makeup gas flow (He)	0.5 L min^−1^	Laser pulse duration	5 ns
Makeup gas flow (N)	0.006 L min^−1^	Laser power	0.02 mJ
		Laser fluence	1.024 J/cm^2^
**Olfactory bulb scans**			
Laser beam diameter	50 µm		
Scan speed	50 µm s^−1^		
Distance between lines	20 µm

**Table 4:** Experimental parameters used for LA-ICP-MS experiments.

Analyte	Mass (amu)	Isotopic abundance	Scan mode	Dwell time per amu (ms)
Al	26.9815	100%	Peak hopping	95
Mn	54.9381	100%	Peak hopping	180
Zn	65.926	27.81%	Peak hopping	180
Zn	67.9249	18.57%	Peak hopping	95
Cu	62.9298	69.1%	Peak hopping	95
Cu	64.9278	30.9%	Peak hopping	95
Fe	53.9396	5.82%	Peak hopping	40
Fe	56.9354	2.19%	Peak hopping	40
Ca	42.9588	0.145%	Peak hopping	95

### LA-ICP-MS data processing and heat map creation

Data were analyzed using Microsoft Excel 2010 and heat maps were created in R v2.15.2. In Excel, ICP-MS data in counts per second (cps) were manually aligned using representative images of each section. Matrix-matched standard data were removed for later use before counts were split out into each isotope and background corrected. ^63^Cu and ^66^Zn raw counts were converted into absolute concentrations using the slope of the matrix-matched-standard results, while ^57^Fe counts were converted into relative levels using the average ^57^Fe counts from the blank (no added metals) matrix-matched standard, as numerous attempts to create iron-spiked standards were unsuccessful. Data were then smoothed in Excel using mean-filter smoothing to reduce pixelation, and heat maps were created using R v2.15.2 (code is given in Suppl. Figure 2) and resized in Adobe Photoshop CS6.

### Immunohistochemistry

For all sections that had been scanned using LA-ICP-MS, immunohistochemistry was performed. Immunohistochemistry was also performed on adjacent, naïve sections to account for LA-ICP-MS-induced artefacts. Sections that were 12 or 30 µm thick were fixed for 10 min in 15% formalin, rinsed, blocked in 10% normal goat serum, and rinsed again. Sections that had been scanned for LA-ICP-MS were then subjected to antigen retrieval because tissue had been air dried for several weeks before immunohistochemistry was performed. These sections were heated and then cooled for 2 h in a pressure cooker in Tris-EDTA (pH 9.0). All sections were then incubated overnight at 4 °C in primary antibody diluted in immunobuffer serum (rabbit alpha-synuclein phosphorylated at serine 129, 1:3,000, Abcam ab51253; mouse protein gene product 9.5, 1:1,000, Abcam ab8184; chicken glial fibrillary acidic protein, 1:4,000, Abcam ab4674). Sections were then rinsed thoroughly and incubated at room temperature for 3 h in a secondary antibody conjugated to a fluorophore (Alexa Fluor 488 goat anti-mouse IgG, Alexa Fluor 594 goat anti-rabbit IgG, or Alexa Fluor 647 goat anti-chicken IgG; all diluted at 1:400 in immunobuffer serum). After being rinsed thoroughly with PBS, sections were incubated for 5 min in Hoechst (Molecular Probes; diluted at 1:20,000 in phosphate-buffered saline). Following rinsing, sections were either coverslipped with ProLong Gold Antifade mountant (ThermoFisher) or were further stained using the immersion neoTimm method (below).

Fluorescent micrographs were taken on a Zeiss Axio MetaSystems VSlide slide scanner using a 20x objective (0.9 NA), while confocal micrographs were taken on an Olympus FV1000 confocal microscope using a 40x objective (NA 1).

### Histology

The immersion neoTimm method^50^ was used to visualize free and loosely bound zinc in 12 µm sections of olfactory bulb and tract. All glassware was washed overnight with Farmers solution (5% potassium ferricyanide and 5% sodium thiosulphate) before use. Tissue sections were incubated in neoTimm solution (0.1% sodium sulfide and 3% glutaraldehyde in 0.1 M phosphate buffer) for 72 h at 4 °C. Slides were rinsed very thoroughly in 0.1 M phosphate buffer and incubated in autometallography developer solution (0.12% silver lactate, 0.85% hydroquinone, and 20% gum arabic in a sodium citrate buffer) for 1 h at 27 °C. Sections were then immersed for 10 min in stop buffer (5% sodium thiosulphate) and rinsed in water. Slides that had previously been immunohistochemically labelled were then coverslipped with ProLong Gold, while naïve sections were either processed for transmission electron microscopy or were dehydrated, counterstained with cresyl violet, and coverslipped with DPX mounting medium for imaging under a light microscope. No staining was seen in negative control conditions, where sodium sulfide was omitted from the neoTimm solution.

Modified Perls and Turnbull methods (adapted from^103^) were used to visualize iron in 12 µm formalin-fixed sections of olfactory bulb and tract. The Perls stain allows mostly ferric iron to be observed, and the Turnbull method stains ferrous iron^49^; both stains can be enhanced using 3,3’-diaminobenzidine (DAB) for a more sensitive method^104^. Endogenous peroxidases were blocked by a 20-min incubation in a 50% methanol, 1% H_2_O_2_ solution. Sections were then rinsed in distilled water before a 30-min incubation in filtered Perls (5% potassium ferrocyanide in 10% concentrated HCl) or Turnbull (10% potassium ferricyanide in 0.5% concentrated HCl) solution. Following thorough rinsing, sections were incubated in DAB solution (0.05% DAB, 0.01% H_2_O_2_ in 0.1 M phosphate buffer) for 15 min. Sections were then rinsed, dehydrated, counterstained with cresyl violet, and coverslipped using DPX mounting medium. No staining was seen in negative control conditions, where potassium ferrocyanide or potassium ferricyanide was omitted from the staining solution.

Light micrographs were taken on a Zeiss Axio MetaSystems VSlide slide scanner using a 20x objective (0.9 NA).

### Transmission electron microscopy

For the electron microscope studies, 12 µm sections that had been labelled with neoTimm solution or control solution (where sodium sulfide was omitted from the neoTimm solution) had the anterior olfactory nucleus regions carefully dissected out. This tissue was postfixed for 1 h at 4 °C in 1% osmium tetroxide in 0.1 M phosphate buffer, dehydrated through a series of ethanols and acetone, and infiltrated with increasing ratios of resin (Taab 812) in acetone. Following an overnight resin treatment, the tissue was flat embedded in fresh resin and cured for 48 h at 60 °C. 80 nm sections were cut on an ultramicrotome (Leica Ultracut UCT Ultramicrotome), collected on copper grids, and stained with aqueous uranyl acetate and Reynolds lead citrate before imaging with a Tecnai G2 Spirit Twin transmission electron microscope.

## Electronic supplementary material

Supplementary Material
