# Supplementary material for: Metal concentrations and distributions in the human olfactory bulb in Parkinson’s disease
Source: Sci Rep. 2017 Sep 5;7:10454. doi: 10.1038/s41598-017-10659-6 (PMC5585381; doi:10.1038/s41598-017-10659-6)
Supplement: Supplementary file 1 — Supplementary Material [file 41598_2017_10659_MOESM1_ESM.pdf]

Metal concentrations and distributions in the human olfactory bulb in Parkinson's disease..

Bronwen Gardner, Birger V. Dieriks, Steve Cameron, Lakshini H. S. Mendis, Clinton Turner, Richard L. M. Faull, Maurice A. Curtis*

Corresponding author: Maurice Curtis; affiliation: Centre for Brain Research and Department of Anatomy with Medical Imaging, University of Auckland; email: m.curtis@auckland.ac.nz

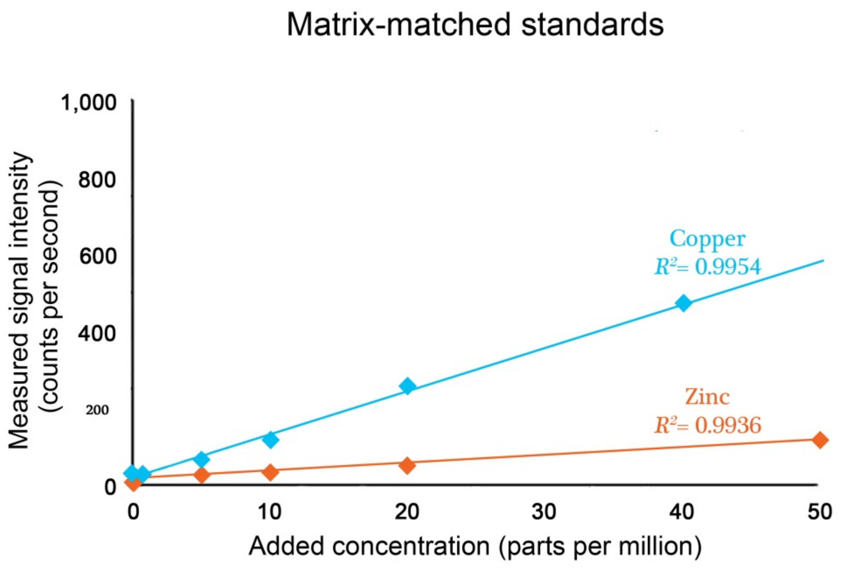

Supplementary Figure 1: Graph showing the linear relationship between added metals and measured signal intensity from LA-ICP-MS.

P:\LA-ICP-MS\Good heat map script purple red white.R - R Editor

```
Map <- read.csv("P:\\LA-ICP-MS\\2015.4.30 OFB 30.4.15\\For Maps Smoothed\\Mn 8.csv", sep=",")
Map_matrix <- data.matrix(Map)
Colorme <- colorRampPalette(c("black", "dark blue", "blue", "purple", "red", "orange", "yellow", "light yellow", "white"))(n = 1000)
Map_heatmap <- heatmap(Map_matrix, Rowv=NA, Colv=NA, col = Colorme, scale="none", margins=c(5,5))

Colorme <- colorRampPalette(c("black", "white"))(n = 10000)
Map_heatmap <- heatmap(Map_matrix, Rowv=NA, Colv=NA, col = Colorme, scale="none", margins=c(5,5))
```

P:\LA-ICP-MS\Colour bar for good heat map purple red white.R - R Editor

```
color.bar <- function(lut, min, max=-min, nticks=2, ticks=seq(min, max, len=nticks), title='') {
    scale = (length(lut)-1)/(max-min)
    dev.new(width=1.75, height=5)
    plot(c(0,10), c(min,max), type='n', bty='n', xaxt='n', xlab='', yaxt='n', ylab='', main=title)
    axis(2, ticks, las=1)
    for (i in 1:(length(lut)-1)) {
      y = (i-1)/scale + min
      rect(0,y,10,y+1/scale, col=lut[i], border=NA)
    }
}

Colorme <- colorRampPalette(c("white", "light yellow", "yellow", "orange", "red", "purple", "blue", "dark blue", "black"))(n = 1000)
lut = rev(Colorme)
color.bar(lut, 0, 45)
```

Supplementary Figure 2: R code used to create heat maps from LA-ICP-MS data
